# Genome-wide association study of sarcopenia index reveals sex-stratified genetic architecture

**DOI:** 10.1186/s13293-026-00973-y

**Published:** 2026-08-25

**Authors:** Yumeng Mu, Binzhi Liao, Mengliang Luo, Kaifeng Lu, Huaxin Tang, Mao Nie, Xianding Sun

**Affiliations:** Department of Orthopedic Surgery, The Second Affiliated Hospital of Chongqing Medical University, Chongqing, 400010 China

**Keywords:** Sarcopenia, Genetic association, Multi-omics, Aging-related mechanisms, Sex-stratified analysis

## Abstract

**Background:**

The sarcopenia index (SI), defined as the ratio of serum creatinine to cystatin C, is a proposed biomarker of muscle mass and sarcopenia, yet its genomic basis and genetic architecture remain largely unexplored.

**Methods:**

We performed combined-sex and sex-stratified genome-wide association studies of SI in the UK Biobank. We examined the overlap between SI-associated loci and loci previously reported for sarcopenia-related traits. We assessed sexually dimorphic effects and gene–sex interactions, performed fine-mapping, and conducted credible gene prioritization, motif and transcription factor binding enrichment, gene-set enrichment, linkage disequilibrium score regression, and cross-phenotype colocalization.

**Results:**

We identified 774 unique independent SI-associated loci across all analyses, with 747 detected in the combined-sex GWAS, 283 in the male-stratified GWAS, and 311 in the female-stratified GWAS; 367 of these loci had not been previously reported for conventional sarcopenia-related traits. Sex-stratified analyses highlighted the rs1145093–chr15q21.1–GATM region, where CARMA identified sex-differentiated causal variants. We prioritized 17 male-biased and 11 female-biased credible genes. Enrichment analyses implicated androgen receptor and GATA4 in males, and ESR1 and MYOD1 in females. Enrichment revealed shared pathways involving inflammation, cellular stress, and aging-related processes. LDSC showed inverse genetic correlations between SI and heart failure (rg = −0.19, *p* = 2.30 × 10^− 9^) and metabolic syndrome (rg = −0.12, *p* = 8.49 × 10^− 8^), and a positive correlation with chronic kidney disease. Compared with female SI, male SI exhibited two additional loci showing colocalization with four metabolic traits.

**Conclusions:**

These findings clarify the genetic architecture of SI and reveal sex-dependent mechanisms underlying sarcopenia, supporting precision risk assessment and targeted interventions.

**Supplementary Information:**

The online version contains supplementary material available at https://doi.org/10.1186/s13293-026-00973-y.

## Highlights

Sex-stratified GWAS identified 247 loci reaching genome-wide significance in only one sex among 774 independent SI-associated loci, of which 59 showed stronger associations in the corresponding sex-stratified GWAS than in the combined-sex analysis.

The rs1145093–chr15q21.1–GATM region showed a prominent sex-dependent association via sex-stratified analyses.

Sex-stratified functional annotation pointed to male-enriched androgen receptor and GATA4, female-enriched ESR1 and MYOD1, and common biological processes related to inflammation, stress response, and aging.

Cross-phenotype colocalization identified rs1229984 at chr4q23 and rs9817452 at chr3q25.31 as loci showing male-stratified colocalization with four metabolic traits.

**Supplementary Information:**

The online version contains supplementary material available at https://doi.org/10.1186/s13293-026-00973-y.

## Abstract (de)

The sarcopenia index, a proposed biomarker of muscle mass and sarcopenia, is calculated from two routine blood markers including creatinine and cystatin C. This index may reflect muscle health, but little is known about how a person’s genes influence it, or whether these genetic influences differ between women and men. In this study, we used data from the UK Biobank to study genetic factors linked to the sarcopenia index. We analyzed women and men together, and then separately, to look for both shared and sex-stratified genetic patterns. We found many genetic regions linked to the sarcopenia index, including several that had not been reported before. Some genetic signals were seen mainly in women, while others were seen mainly in men. One important region near a gene called GATM showed especially strong sex-dependent patterns. Our findings also suggested that different hormone-related and muscle-related biological processes may be more important in women and men. For example, signals related to androgen activity were more prominent in men, while estrogen-related signals were more prominent in women. We also found that the sarcopenia index shares genetic links with several long-term health conditions, including heart failure, metabolic syndrome, and chronic kidney disease. Overall, this study improves our understanding of the genetic background of muscle health and highlights the importance of considering sex differences when assessing sarcopenia risk and developing prevention strategies.

## Background

Sarcopenia, an age-related disease characterized by the progressive decline in skeletal muscle mass and function, substantially lowers the quality of life in older adults while elevating the risks of falls, disability, and mortality [1, 2]. In recent years, the sarcopenia index (SI), defined as the ratio of serum creatinine to cystatin C, has emerged as a novel biomarker, garnering significant attention in sarcopenia research due to its non-invasive nature and ease of integration into routine clinical practice [3, 4]. Previous studies have demonstrated a robust correlation between SI and conventional markers such as grip strength and muscle mass [5, 6]. Moreover, SI predicts sarcopenia with greater accuracy than creatinine or cystatin C alone, highlighting its superior potential for clinical application. These advantages make SI a valuable tool for elucidating the underlying mechanisms of sarcopenia, offering fresh insights into associated genetic and therapeutic research.

Genome-wide association studies (GWAS) offer a powerful framework for elucidating the genetic architecture of complex traits by linking genetic variants to phenotypic variation [7]. In sarcopenia research, GWAS have identified variants associated with muscle strength, appendicular lean mass (ALM), and gait speed [8]; however, most studies have focused on these conventional phenotypes, with limited investigation of emerging biomarkers. Therefore, GWAS targeting novel indices may provide a more comprehensive understanding of the genetic basis of sarcopenia. Given the pronounced sex differences in muscle mass and strength, sex-stratified GWAS analyses are essential for uncovering genetic variants associated with SI and elucidating the genetic mechanisms underlying sex-differentiated manifestations of sarcopenia, thereby informing precision intervention strategies [9]. The UK Biobank (UKB), a large-scale prospective cohort comprising phenotypic and genotypic data from approximately 500,000 participants, provides an opportunity for conducting high-quality GWAS [4, 8, 10].

**Fig. 1:**
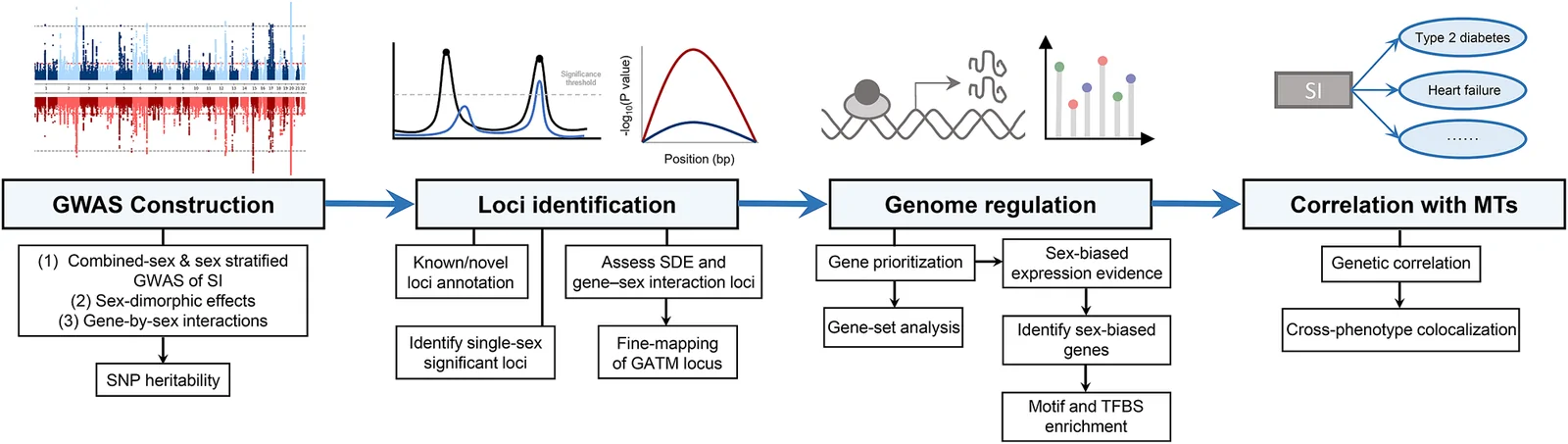
Study workflow. We performed combined-sex and sex-stratified GWAS of SI, followed by identification of associated loci and fine-mapping. Functional analyses included gene prioritization, gene-set analysis, sex-biased gene identification, motif and TFBS enrichment. Finally, genetic correlation and cross-phenotype colocalization were conducted to assess shared genetic architecture with metabolic diseases

In our study (Fig. 1), we utilized UKB data to perform GWAS analyses focusing on the serum creatinine to cystatin C ratio, with the aim of identifying SI-associated genetic variants. Additionally, we performed sex-stratified GWAS to explore the genetic mechanisms contributing to sex differences in sarcopenia. These findings enhance our understanding of the genetic architecture of sarcopenia and its sex-stratified manifestations, offering novel insights for risk assessment and precision intervention strategies.

## Methods

### Ethics

Our study was conducted in strict accordance with all requisite ethical standards. Data were obtained from an approved UKB application (application number 624933). The UKB [11] has received ethical approval from the National Health Service North-West Centre Research Ethics Committee (reference number 11/NW/0382). All participants provided written informed consent upon enrollment in the UKB program, and the study adheres to the principles outlined in the Declaration of Helsinki.

### Study population

We analyzed UKB data following standard quality control procedures. Participants with concordant self-reported and genetic sex were included. Analyses were restricted to white British participants, excluding individuals with sex chromosome aneuploidy, excessive relatedness, or genotyping quality outliers. Participants were required to have complete data on serum creatinine, cystatin C, BMI, and smoking status. After quality control, 380,734 individuals, including 205,991 females and 174,743 males, were available for analysis. To ensure comparable statistical power, male and female participants were matched by age and genetic similarity. Matching was initially performed within a 5-year age range and extended to 10 years if necessary. Genetic similarity was assessed using principal components, and the closest male–female pairs were retained. Ultimately, 349,486 participants were included in the final analysis, comprising 174,743 females and 174,743 males.

### Genome-wide association analyses

GWAS were conducted using imputed genotype data. Variants were filtered based on a minor allele frequency (MAF) ≥0.001, Hardy–Weinberg equilibrium *p* ≥ 1 × 10^− 6^, and an imputation score ≥ 0.3, yielding 14.66 million single-nucleotide polymorphisms (SNPs). SI was calculated using serum creatinine and cystatin C levels from UK Biobank data fields 30,700 and 30,720, respectively. All models were adjusted for age, age squared, the first 20 principal components, genotyping array, smoking status, and body mass index. Both sex-stratified and combined-sex analyses were performed, with sex included as a covariate in the combined-sex models. Analyses were conducted using REGENIE. A whole-genome regression model was first fitted using directly genotyped variants to account for population structure and relatedness, followed by association testing of imputed variants. Only common variants with MAF ≥ 0.01 were retained for downstream analyses.

### SNP heritability and partitioned S-LDSC

Using the European 1000 Genomes Phase 3 (1kGP3) panel [12] as the reference, SI heritability was estimated by linkage disequilibrium score regression (LDSC; https://github.com/bulik/ldsc) [13]. Stratified LDSC (S-LDSC) [14] was applied to assess partitioned heritability across genomic functional annotations. Single-nucleotide variants (SNVs) were grouped into 53 functional categories as defined by Finucane et al. [14], and heritability enrichment was evaluated using precomputed LD scores and annotation datasets, with Bonferroni correction applied.

### Identification of independent loci and single-sex significant loci

Genome-wide significant variants (*p* < 5 × 10^−8^) were extracted from the combined-sex, male-stratified, and female-stratified GWAS results. Independent index SNPs were identified using PLINK [15] by LD pruning (r^2^ > 0.1 within ±500 kb), and proxy SNPs were defined as variants with r^2^ > 0.2. For each independent locus, sex-stratified significance was evaluated at the locus level by considering both the index SNP and its proxy SNPs. A locus was considered significant in a given sex if either the index SNP or any of its proxy SNPs reached genome-wide significance in the corresponding sex-stratified GWAS. Loci reaching genome-wide significance in only one sex-stratified GWAS were operationally defined as single-sex significant loci, including male-only and female-only significant loci. Loci that did not meet this definition were classified as non-single-sex significant loci, including loci significant in both or neither sex-stratified GWAS.

### Annotation of previously defined sarcopenia-related loci

To annotate previously reported sarcopenia-related loci, we compared SI-associated index SNPs and their proxy SNPs with genome-wide significant variants reported for ALM [16], handgrip strength (HGS; OpenGWAS ID: ukb-b-7478), and walking pace (WP) [17]. SI loci were classified as previously reported if the index SNP or any corresponding proxy SNP met either of the following criteria: (1) LD with known ALM-, HGS-, or WP-associated variants (r^2^ > 0.2 within ±500 kb); or (2) physical proximity within 50 kb of known ALM-, HGS-, or WP-associated variants, irrespective of LD. Loci meeting neither criterion were considered novel.

### Defining sexually dimorphic effects (SDEs)

To identify genetic effects that differed between males and females, effect estimates for each SNP from male- and female-stratified GWAS were compared by calculating the SDE z-score. The null hypothesis assumed equality of effect sizes between sexes, with SDEs reflecting differences in the magnitude or direction of SNP effects. The SDE z-score [18] was computed as: ${{{\rm{Bet}}{{\rm{a}}_{{\rm{female}},i}} - {\rm{Bet}}{{\rm{a}}_{{\rm{male}},i}}} \over {\sqrt {{\rm{SE}}_{{\rm{female}},i}^2 + {\rm{SE}}_{{\rm{male}},i}^2} }}$, where Beta_female,I_ and Beta_male,i_ respectively denote the regression coefficients from female and male GWAS models, and SE_female,i_ and SE_male,i_ represent their corresponding standard errors. Two-tailed *p* values were then derived from SDE z-scores to represent the significance of sex differences in SNP effects. Loci were annotated using FUMA [19], lead SNPs reaching *p* < 5 × 10^−8^ were considered genome-wide significant SDE loci, whereas SNPs with *p* < 1 × 10^−5^ were reported as suggestive SDE signals for exploratory prioritization only. Accordingly, loci identified by the SDE analysis were interpreted as showing formal evidence of sex-differentiated genetic effects.

### Genotype–sex interaction analysis and position mapping

Quality control for the sex interaction analysis followed that of the SI GWAS. Sex–genotype interaction analyses were performed using GEM [20], with relatedness estimated by GCTA and restricted to unrelated samples. Covariates were consistent with those used in the GWAS, and the following model was implemented to evaluate interactions: SI ∼ G × sex + G + sex + age + age^2^ + BMI + PC1∼20 + genotyping array + smoking. Common variants (MAF > 0.01) were retained. Interaction effects were evaluated using two-sided *p* values, with loci identified using FUMA. Strong SNP–sex interactions were defined using a Bonferroni-corrected genome-wide significance threshold, while *p* < 10^− 5^ was considered suggestive. Gene-level association analysis was conducted using MAGMA [21].

### Fine mapping of the chromosome 15q21.1-GATM region

Fine-mapping analyses were performed for significant loci identified in the genotype–sex interaction analysis. Regions extending ±250 kb around each locus were analyzed using PolyFun [22] and CARMA [23]. PolyFun was used to generate functionally informed prior causal probabilities for candidate SNVs, which were subsequently incorporated into CARMA to identify putative causal variants. Credible sets were constructed by aggregating posterior inclusion probabilities (PIPs) until a cumulative PIP of 99% was reached, and variants within these sets were considered potential contributors to the observed associations.

### Gene prioritization

Gene prioritization was performed for regions extending ±500 kb around independent loci using mBAT-combo [24] and colocalization analyses. mBAT-combo integrates mBAT and fastBAT statistics via a Cauchy combination method to test gene-level associations while accounting for complex LD structures. Bonferroni correction was applied, and genes with adjusted P_mBAT_ < 0.05 were considered associated with SI. Colocalization analysis was conducted using the coloc package with an approximate Bayes factor framework to evaluate whether SI and gene expression shared causal variants. Skeletal muscle eQTL data from GTEx v8 [25] were used, and genes with posterior probability for a shared causal variant (PP.H4 > 0.5) were prioritized.

### Sex-biased gene expression effects

The GTEx v8 project performed sex-biased analyses on muscle skeletal tissues, encompassing a total sample size of 816 individuals, including 551 males and 265 females. The MASH [26] and voom-limma [27] were utilized to identify sexually differentially expressed genes. We compared genes exhibiting significant sex-biased expression in muscle skeletal tissues (local false sign rate ≤ 0.05, with effects differing from zero at a 95% CI) against those associated with SI.

### Identification of sex-biased genes

For each sex-stratified GWAS, genes attaining significance in both gene prioritization methods were designated as sex-stratified credible genes. To ascertain sex-biased genes, the following criteria were applied: (1) In mBAT analysis, significance was observed exclusively in one sex, with a D_mbat_ value exceeding 2 (D_mbat_ = |-log_10_(p_male_) - (-log_10_(p_female_)) |); (2) In colocalization analysis, significance was confined to one sex, with the PP.H4 for that sex differing from the other by more than 0.3; (3) The gene demonstrated sex-biased expression in muscle skeletal. Genes meeting all three criteria were classified as sex-biased genes.

### Motif enrichment

For sex-biased genes, nuclear receptor (NR) motif enrichment analysis was conducted to determine whether promoter regions (defined as − 1000 to +1000 base pairs relative to the transcription start site) were enriched for specific NRs. HOMER [28] was employed to analyze sequences of 8 bp, 10 bp, 15 bp, and 20 bp in length, with background sequences automatically matched by HOMER for reference. Motif enrichment significance was evaluated using both uncorrected *p* values and Bonferroni-corrected *p* values.

### Transcription factor binding site (TFBS) enrichment

Based on the LOLA database [29], TFBS enrichment was performed using UniBind [30, 31]. Comparisons involved male-only significant loci, female-only significant loci, and single-sex significant loci. Regions extending ±500 kb around each locus were tested against a background set of 21,031 genes expressed in skeletal muscle from GTEx v8. Enrichment was assessed using a one-tailed Fisher’s exact test, and TFs were ranked according to their nominal *p* values, with the top 10 TFs reported for each comparison.

### Gene-set analysis

To elucidate the biological functions of SI-associated genes, pathway enrichment analyses were performed on credible genes for male, female, and combined sexes, uncovering underlying biological processes and molecular mechanisms. These analyses were based on the KEGG [32], GO [33], Reactome [34], and WikiPathways [35] databases, utilizing the clusterProfiler and ReactomePA R packages. Enriched pathways with q values < 0.05 were deemed closely associated with SI.

### Genetic correlations with metabolic diseases

Genetic correlations between the SI across three sex categories and 17 metabolic diseases were estimated using LDSC, with the 1kGP3 as the reference. Results were reported as genetic correlation coefficients (rg, range −1 to 1), with significance assessed using Bonferroni correction. Significant correlations were interpreted as evidence of shared genetic architecture between SI and metabolic diseases.

### Cross-phenotype colocalization analysis

To investigate the shared genetic architecture between SI and metabolic traits, colocalization analysis was conducted on SI-associated loci using the abf approach in the coloc package [36]. Four hypotheses were evaluated: H0, absence of causal variants for either SI or the metabolic trait in the region; H1/H2, presence of a causal variant for only one trait; H3, causal variants for both traits that are distinct; H4, a shared causal variant for both traits. We focused on the PP.H4, with PP.H4 > 0.7 indicating strong evidence of shared causal variation. Colocalization was performed separately using male- and female-stratified SI GWAS summary statistics, and the results were compared across sexes.

## Results

### Genome-wide association analyses of SI

**Fig. 2:**
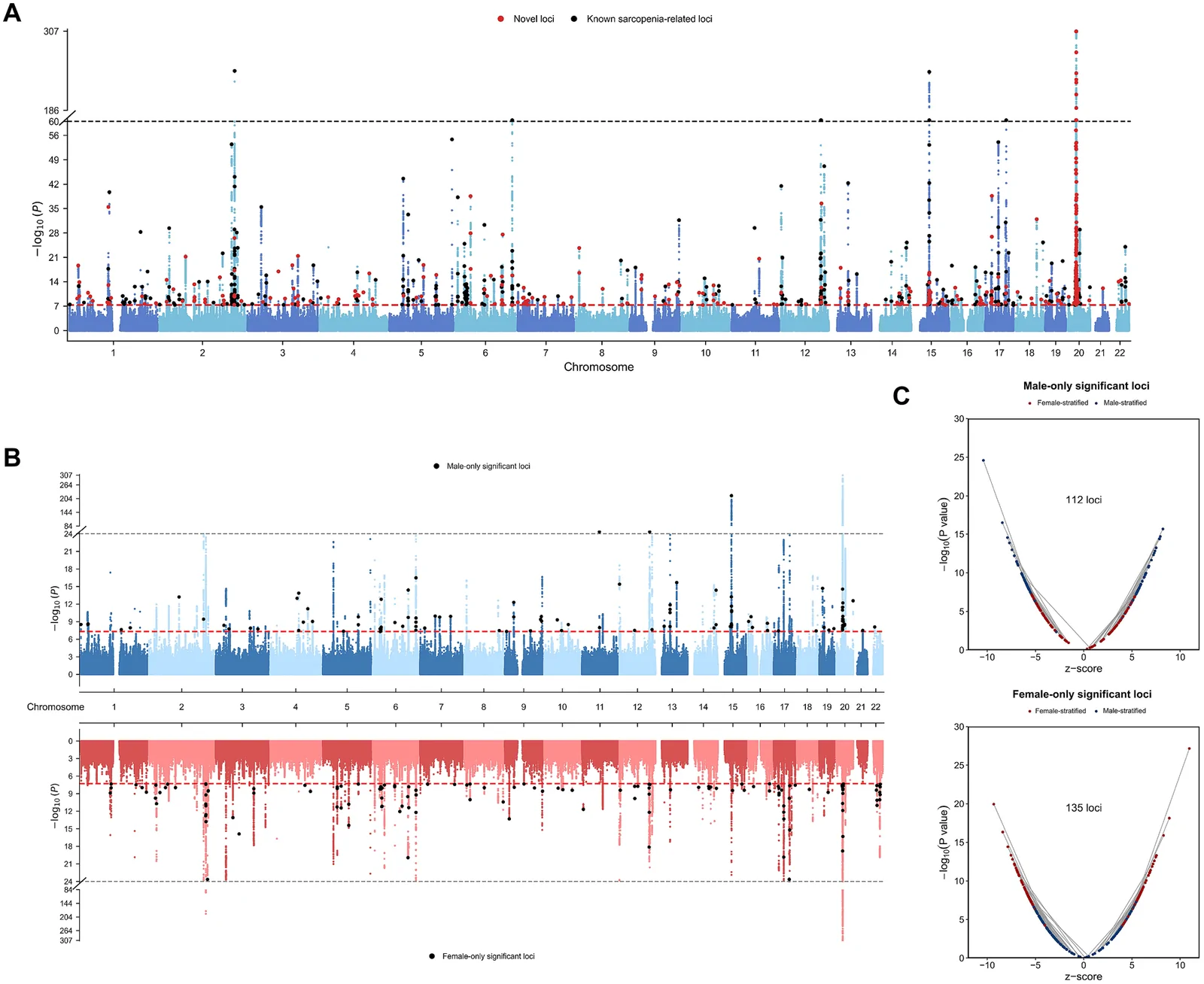
Sex differences in the complex genetic architectures of SI. **A** Manhattan plot displaying combined-sex GWAS results for SI. Red solid dots indicate novel loci, and black solid dots indicate previously reported sarcopenia-related loci. **B** Miami plot displaying male- (blue) and female-stratified (red) GWAS results for SI. Black dots indicate single-sex significant loci, including male-only significant loci in the upper panel and female-only significant loci in the lower panel. **C** Volcano plot of LD-pruned loci reaching genome-wide significance only in the male- or female-stratified GWAS. Each line connects corresponding loci between sexes. Longer lines indicate larger differences in effect sizes, with horizontal and vertical distances representing directional differences and statistical significance, respectively

Using REGENIE, we conducted GWAS of SI, including sex-stratified GWAS with matched sample sizes to characterize sex-stratified association patterns. After filtering rare variants, 8.16 million variants were retained for downstream analyses. LDSC estimated the heritability of SI to be 16.17% in the combined-sex analysis, and 17.83% and 18.82% in males and females, respectively (Figure S1B). We identified 774 unique independent SI-associated loci across all analyses, with 747 detected in the combined-sex GWAS, 283 in the male-stratified GWAS, and 311 in the female-stratified GWAS. (Figs. 2A and 2B). Among loci detected in the sex-stratified GWAS, 135 were female-only significant loci and 112 were male-only significant loci (Table S1). Additionally, three loci exhibited opposite effect directions between sexes. Among these single-sex significant loci, 59 of 247 loci (23.9%) showed stronger associations in sex-stratified GWAS than in the combined-sex analysis, including 23 of 112 male-only significant loci (20.5%) and 36 of 135 female-only significant loci (26.7%). Furthermore, 25 of 247 loci (10.1%) reached genome-wide significance only in sex-stratified analyses, comprising 8 of 112 male-only significant loci (7.1%) and 17 of 135 female-only significant loci (12.6%), but not in the combined-sex analysis. The Pearson correlation coefficient between effect allele frequencies in females and males across significant loci was 0.99, indicating that sex-related heterogeneity was not driven by differences in allele frequencies. Further S-LDSC analysis exhibited that SI of different sex types keep highly consistent in partitioned heritability. Combined-sex, male-stratified and female- stratified SI showed significant associations in seven functional regions such as super enhancer, H3K27ac and conserved. However, the UTR 3 and repressed regions were merely significant in female and combined-sex, indicating that these regions may play specific roles in female SI (Figure S2 and Table S2).

### Annotation of previously unreported loci

We next assessed whether SI-associated loci overlapped with genome-wide significant loci previously reported for ALM, HGS, or WP. Among them, eight loci overlapped with all three traits (rs11060406, rs11666808, rs17207225, rs2763981, rs35325270, rs6120748, rs6142137, and rs71301804), 68 overlapped with two traits, and 331 overlapped with one trait. The remaining 367 loci (47.4%) showed no overlap with previously reported ALM-, HGS-, or WP-associated loci and were therefore classified as novel (Table S3). These findings indicate that SI captures both known sarcopenia-related genetic signals and a substantial number of previously unreported loci (Table 1)

**Table 1:** Missense variants at SI-associated loci not previously reported for sarcopenia-related traits. Variants are categorized as male-only significant, female-only significant, or non-single-sex significant, according to their genome-wide significance patterns in the sex-stratified GWAS. For each SNP, genomic position, alleles, effect allele frequency, mapped gene, and corresponding amino acid change are provided. Association statistics (β, se, and *p* value) are shown for the combined-sex, male-stratified, and female-stratified analyses

SNP	Chromosome	Position	A1	A2	Freq	Gene	AA	Combined-sex GWAS			Male-stratified GWAS			Female-stratified GWAS		
								β	se	p value	β	se	p value	β	se	p value
*Male-only significant*																
rs78444298	1	184672098	A	G	0.02	EDEM3	Pro746Ser	0.81	0.11	2.53E-13	0.96	0.17	1.14E-08	0.71	0.15	1.53E-06
rs1229984	4	100239319	C	T	0.98	ADH1B	His48Leu	−0.71	0.10	5.02E-12	−1.15	0.15	1.06E-13	−0.27	0.14	5.55E-02
rs34130495	6	160560824	A	G	0.03	SLC22A1	Gly401Ser	−0.51	0.09	2.56E-08	−0.80	0.14	6.86E-09	−0.22	0.12	6.67E-02
rs143378550	9	133069741	A	C	0.03	HMCN2	Ser699Ter	−0.76	0.10	1.42E-14	−0.93	0.15	4.18E-10	−0.54	0.13	3.87E-05
rs34400381	11	65143892	A	G	0.03	SLC25A45	Arg243Cys	−0.95	0.08	3.48E-30	−1.31	0.13	2.52E-25	−0.60	0.11	8.33E-08
rs3783344	14	100817759	T	C	0.03	WARS1	Arg267Pro	0.61	0.09	8.39E-11	0.83	0.14	3.37E-09	0.40	0.13	1.58E-03
rs3814995	19	36342212	T	C	0.31	NPHS1	Glu117Ter	−0.22	0.03	1.87E-11	−0.28	0.05	2.87E-08	−0.18	0.04	2.67E-05
rs12975366	19	54759361	C	T	0.40	LILRB5	Asp247Gly	0.22	0.03	8.08E-13	0.22	0.05	3.18E-06	0.20	0.04	9.00E-07
*Female-only significant*																
rs10997975	10	69933921	A	G	0.50	MYPN	Ser416Asn	−0.22	0.03	2.94E-13	−0.23	0.05	5.02E-07	−0.24	0.04	4.74E-09
rs284859	10	104573017	T	G	0.17	WBP1L	Ala320Thr	−0.30	0.04	1.51E-13	−0.27	0.06	9.69E-06	−0.31	0.05	3.85E-09
rs2277339	12	57146069	G	T	0.10	PRIM1	Asp5Val	−0.27	0.05	4.37E-08	−0.13	0.08	7.92E-02	−0.43	0.07	1.49E-10
*Non–single-sex significant*																
rs34611728	1	113255456	A	C	0.13	PPM1J	Leu213Phe	0.61	0.05	1.83E-40	0.45	0.07	3.66E-11	0.76	0.06	4.48E-36
rs2297792	1	156011444	C	T	0.63	UBQLN4	Ile495Met	−0.18	0.03	6.52E-09	−0.19	0.05	8.87E-05	−0.16	0.04	1.13E-04
rs150330307	1	160160801	C	T	0.03	CASQ1	Met87Thr	−0.53	0.09	9.97E-10	−0.48	0.13	2.15E-04	−0.56	0.12	1.25E-06
rs3850625	1	201016296	A	G	0.12	CACNA1S	Arg1539Cys	−0.53	0.05	5.41E-29	−0.49	0.07	6.48E-12	−0.58	0.06	1.37E-20
rs56219475	2	39241107	A	G	0.01	SOS1	Pro655Leu	−1.09	0.15	1.38E-12	−1.17	0.23	4.79E-07	−1.01	0.20	5.94E-07
rs1047891	2	211540507	A	C	0.32	CPS1	Thr1406Asn	1.11	0.03	5.18E-247	0.92	0.05	1.24E-76	1.29	0.04	1.47E-190
rs13107325	4	103188709	T	C	0.07	SLC39A8	Ala391Ser	0.36	0.06	6.29E-10	0.39	0.09	8.80E-06	0.34	0.08	1.45E-05
rs138373837	5	36219710	T	C	0.02	NADK2	Arg48His	0.55	0.10	3.45E-08	0.68	0.15	8.07E-06	0.43	0.13	1.07E-03
rs9379084	6	7231843	A	G	0.12	RREB1	Asp1171Asn	0.63	0.05	5.58E-39	0.67	0.07	3.37E-20	0.58	0.06	1.94E-19
rs1042140	6	33048640	G	A	0.21	HLA-DPB1	Lys98Ter	0.22	0.04	1.18E-08	0.21	0.06	2.00E-04	0.19	0.05	1.29E-04
rs1935	10	64927823	G	C	0.47	JMJD1C	Glu2535Asp	−0.25	0.03	1.01E-15	−0.25	0.05	8.72E-08	−0.23	0.04	8.15E-09
rs2274224	10	96039597	C	G	0.43	PLCE1	Arg1267Gln	0.20	0.03	3.81E-11	0.25	0.05	6.36E-08	0.16	0.04	1.35E-04
rs8187710	10	101611294	A	G	0.06	ABCC2	Cys1515Tyr	0.37	0.07	1.61E-08	0.47	0.10	3.12E-06	0.29	0.09	8.67E-04
rs3184504	12	111884608	C	T	0.52	SH2B3	Trp262Arg	0.73	0.03	1.34E-126	0.77	0.05	2.84E-63	0.68	0.04	1.86E-63
rs28929474	14	94844947	T	C	0.02	SERPINA1	Glu366Gln	−1.15	0.11	5.31E-26	−1.20	0.16	2.07E-13	−1.10	0.14	2.13E-14
rs113956264	16	1997004	T	C	0.03	RPL3L	Val262Met	−0.81	0.09	2.38E-19	−0.89	0.14	5.60E-11	−0.73	0.12	9.66E-10
rs2549677	16	2162361	G	A	0.10	PKD1	Met1092Thr	0.29	0.05	1.79E-08	0.37	0.08	1.83E-06	0.21	0.07	1.76E-03
rs12923138	16	67233266	C	A	0.41	ELMO3	Lys13Gln	0.17	0.03	3.23E-08	0.20	0.05	2.51E-05	0.15	0.04	2.75E-04
rs8052579	16	81129822	A	G	0.92	GCSH	Ser21Leu	0.35	0.06	3.93E-10	0.44	0.08	1.84E-07	0.26	0.07	4.37E-04
rs34536443	19	10463118	C	G	0.05	TYK2	Pro919Ala	0.41	0.07	2.04E-08	0.46	0.11	2.64E-05	0.35	0.10	2.35E-04
rs17750862	20	23377815	A	G	0.06	NAPB	Asn63Lys	1.48	0.07	1.71E-103	1.52	0.10	8.89E-49	1.39	0.09	2.70E-52

### Sex-biased genetic association

**Fig. 3:**
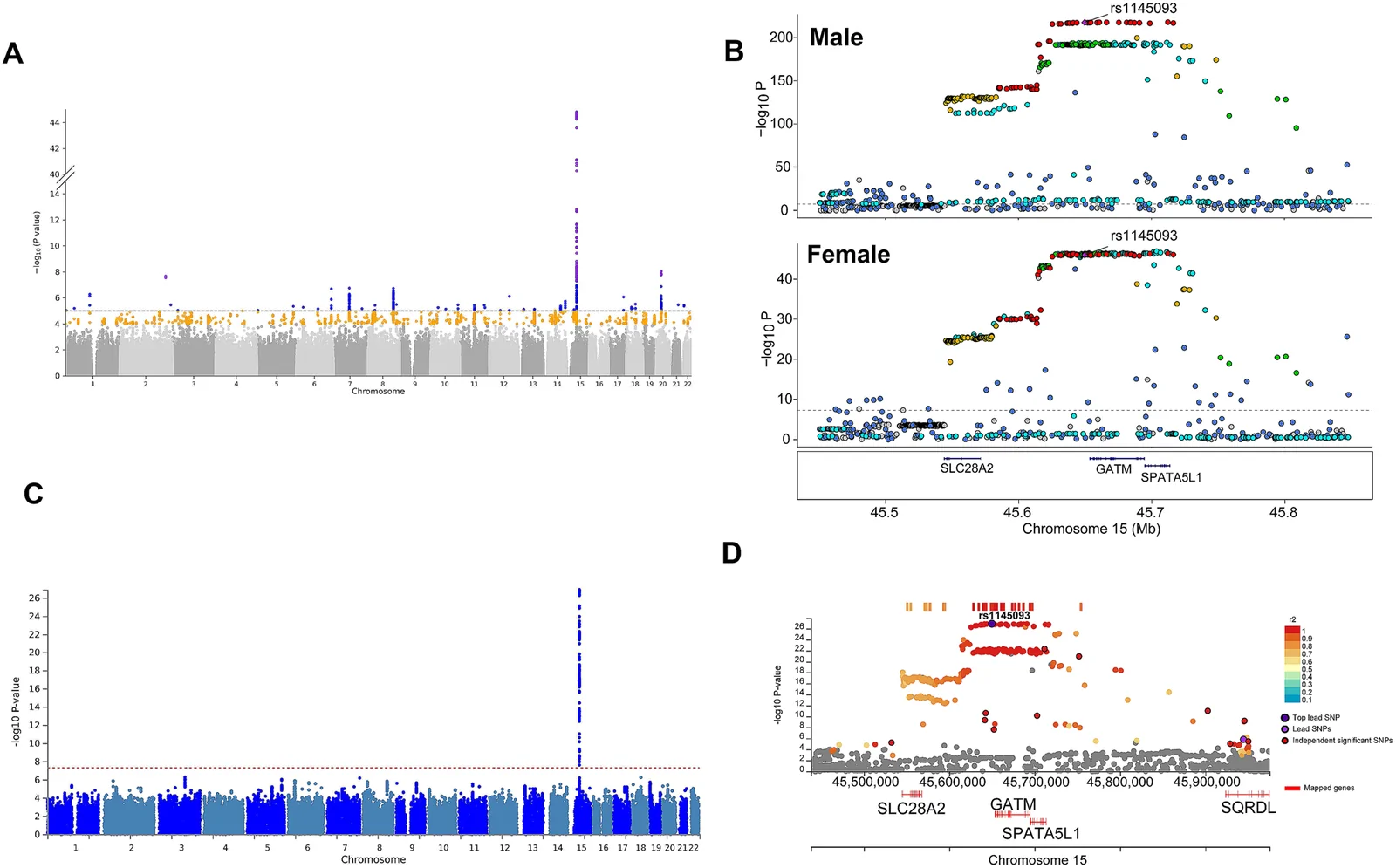
Statistical analysis of sex interactions. **A** Manhattan plot of the SDE analysis results of sex-stratified SI GWAS. Orange, *p* < 10^−4^; blue, *p* < 10^−5^; and purple, *p* < 5 × 10^−8^. **B** The sex-stratified GWAS results for SI at the focal locus on chromosome 15. The upper plot shows association signals in males and the lower plot shows the corresponding results in females, across the 45.5–45.8 mb interval. **C** Regional gene–sex interaction plot of the SI association locus, using linear mixed models adjusted for SI-associated covariates. Red line indicates *p* = 5 × 10^−8^. **D** The gene–sex interaction results for SI at the focal locus on chromosome 15. Association signals for the gene-sex interaction are plotted across the 45.5–45.9 mb interval

To further investigate genetic differences in SI between males and females and to identify loci with opposite effect directions or differential effect sizes across sexes, we performed SDE analysis (Fig. 3A, Table S4, FigureS S3 and S4). Three loci (rs1145093-chr15q21.1-GATM, rs6036481-chr20p11.21-CST3, rs1047891-chr2q34-CPS1) showed significant SDE signals, while an additional 30 loci exhibited suggestive evidence of sex-differentiated effects. Subsequently, we performed gene–sex interaction analysis in an independent set of 251,203 unrelated individuals (Figure S5 and Table S5). This analysis highlighted the locus rs1145093 at chr15q21.1 near GATM again (Fig. 3D), which showed a strong gene–sex interaction signal (*p* = 1.26 × 10^−27^). The other 38 loci Other 38 loci indicated implied associations, among which multiple loci with SDE were validated again. The SDE and gene–sex interaction analyses suggested that several single-sex significant loci, including rs2737205, rs34060476, and rs3808440, may have sex-differentiated effects, potentially through sex-related gene expression or functional pathways.

Given the strong sex-dependent association at the rs1145093–chr15q21.1–GATM locus, we combined PolyFun and CARMA to fine-map a ± 250 kb region around this locus in males and females (Table S6). We focused on SNPs with PIP > 0.99, and there were six for males, five for females, with merely two SNPs (rs675876 and rs68087211) being common signals in two sexes. This indicated that the sex-dependent association at the GATM locus was driven by partial shared causal variants, while there are also sex-specific causal variants, leading to sex differences in the regulation of this region.

### Gene prioritization and identification

To identify credible genes corresponding to each sex-stratified analysis, we extracted loci related to SI and conducted a preliminary screening using mBAT-combo. We combined the GTEx v8 muscle skeletal eQTL data for colocalization (Table S7~ Table S11). Ultimately, we prioritized 108 genes associated with SI in the combined-sex analysis, 65 genes associated with male SI, and 54 genes associated with female SI. Among them, 22 genes were shared across all sex groups, including known genes related to muscle mass such as TSPAN9 and SMAD3, suggesting their involvement in the core pathophysiological process of sarcopenia. To further identify genes showing sex-differentiated expression evidence, we performed a sex-biased gene expression analysis in skeletal muscle tissue using GTEx v8 data (Figure S6). In total, 2,866 genes showed significant sex differences in expression. By intersecting these genes with genes prioritized by mBAT-combo and colocalization analyses, we identified 17 male-biased and 11 female-biased SI-associated genes.

### Genome regulation of SI-associated loci

**Fig. 4:**
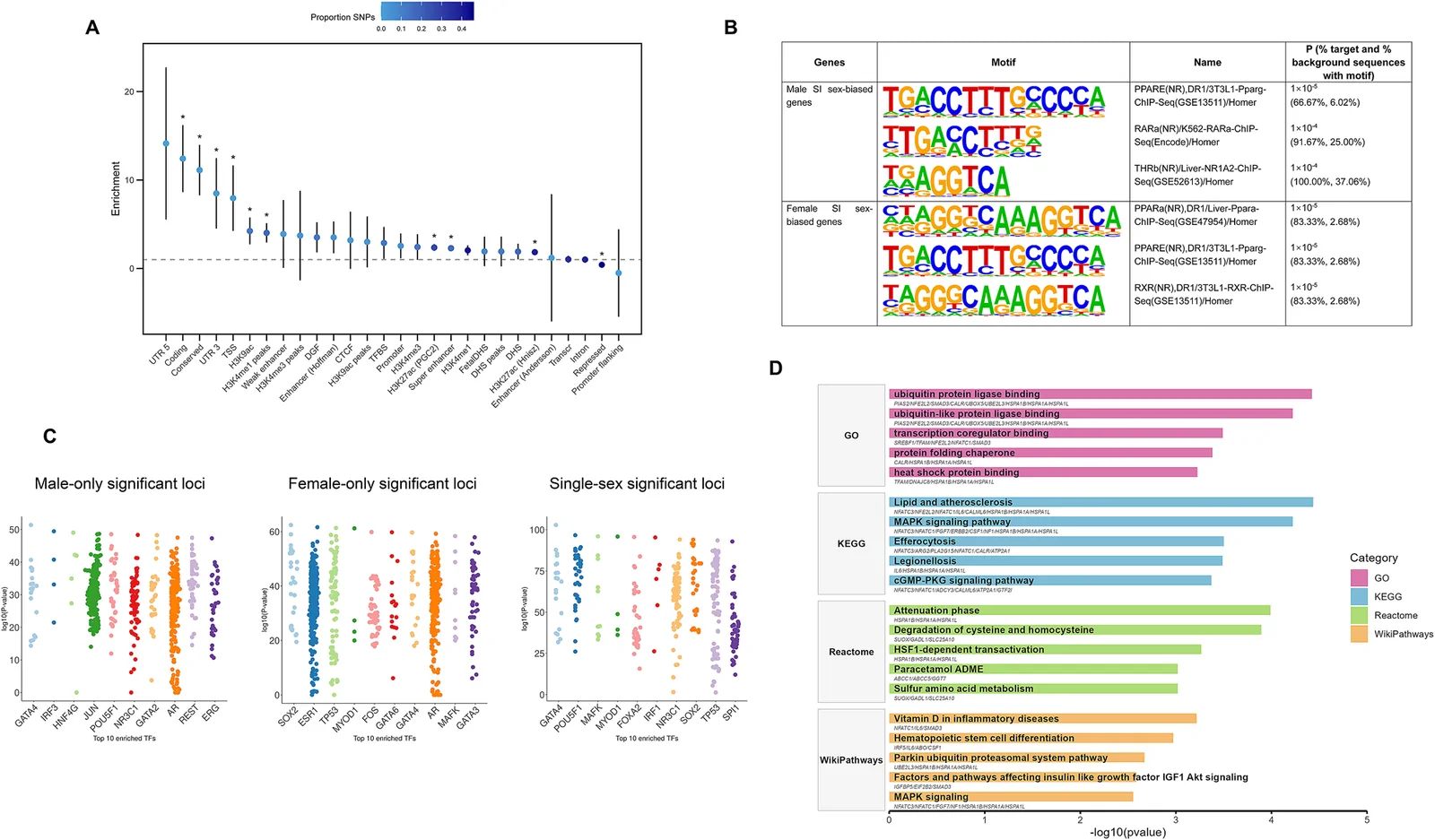
Functional genomic enrichment of shared and sex-stratified SI architectures. **A** The S-LDSC heritability enrichment estimates for SI across 53 genomic functional annotations. Mean enrichment values are plotted for each category, with points colored by the proportion of SNPs assigned to that category and a horizontal dashed line indicating the null expectation of no enrichment. **B** Motif enrichment analysis of NRs in the promoter regions of sex-biased genes identified from sex-stratified GWAS results. **C** Top ten differentially enriched TFBSs from comparisons among male-only significant loci, female-only significant loci, and single-sex significant loci, ranked by uncorrected *p* values. **D** Functional enrichment analysis of credible gene sets across GO, KEGG, Reactome, and WikiPathways categories. Bars represent significantly enriched pathways ranked by −log₁₀(*p* value), with colors indicating database sources

For genes with sex bias, we further conducted an enrichment analysis of known NR motifs in their promoter regions. In the promoter regions of male-biased genes, PPARE, RARα, and THRb motifs were significantly enriched (Fig. 4Band Table S12), highlighting the core role of metabolic and myogenic transcriptional regulation in muscle growth and function. In the promoter regions of female-biased genes, PPARα (NR), PPARE (NR), and Erra (NR) motifs were significantly enriched, suggesting the key role of lipid metabolism in female sarcopenia. In further TFBS analysis, in males, AR and GATA4 were significantly enriched, further emphasizing the role of androgen signaling and the GATA family in muscle growth and anti-apoptosis (Fig. 4C). The enrichment of IRF3 suggests the regulation of inflammation and metabolic homeostasis. In female-only significant loci, ESR1 and MYOD1 were significantly enriched, respectively associated with the protective effects of estrogen signaling on metabolic health and mitochondrial function, as well as the regulation of muscle differentiation. In combined-sex loci, the enrichment of MYOD1 and FOXA2 indicates that muscle differentiation and inflammatory response are common mechanisms of sarcopenia. The differential analysis of single-sex significant loci and shared loci showed that the enrichment of HIF1A and AR suggests sex differences in hypoxia stress and muscle repair, which may affect the susceptibility or progression of sarcopenia.

### Enrichment analysis

Pathway enrichment analysis of credible gene sets across sex categories revealed core biological processes closely associated with the SI (Table S13). Across all analyses, the MAPK signaling pathway and cellular senescence pathway were consistently enriched, indicating that inflammation, cellular stress, and aging represent shared molecular mechanisms underlying sarcopenia. In the combined analysis, pathways related to protein degradation, including ubiquitin-like protein ligase binding, were prominently enriched, highlighting a central role of the ubiquitin–proteasome system in muscle protein breakdown. Sex-stratified analyses revealed distinct biological patterns. In females, enriched pathways included insulin-like growth factor transport and uptake as well as muscle adaptation-related processes, pointing to dysregulation of muscle synthesis and remodeling. In contrast, male-associated gene sets were predominantly enriched in Wnt signaling and Sema4D signal transduction pathways, which are involved in cell migration, regeneration, and development, suggesting a greater dependence on impaired muscle stem cell function and tissue repair capacity. Notably, wound healing and immune-related pathways, such as Th17 cell differentiation, were enriched in both sexes, further supporting a universal contribution of chronic inflammation to the pathophysiology of sarcopenia.

### Genetic correlation with metabolic traits

**Fig. 5:**
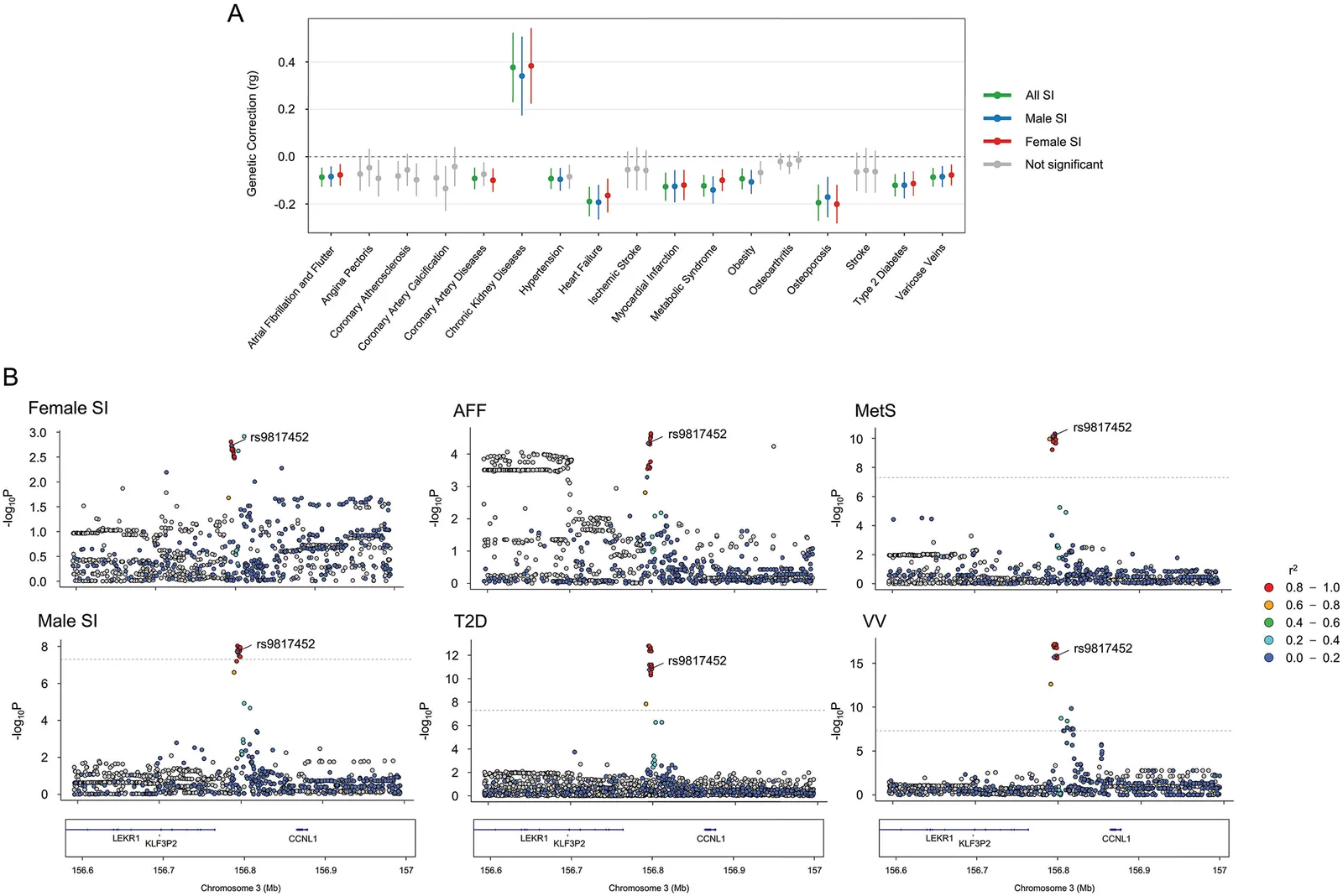
Genetic correlations and cross-phenotype colocalization between SI and metabolic traits. **A** LDSC-estimated genetic correlations (rg) of combined-sex, male-stratified, and female-stratified SI with 17 metabolic diseases. Points represent rg estimates with error bars indicating standard errors. **B** Regional association and colocalization plots for SI and representative metabolic traits. SNP associations are shown as −log₁₀(P) values across genomic positions, with colors indicating linkage disequilibrium (r^2^) with the lead variant

Previous cohort studies have reported associations between the SI and metabolic traits. Using LDSC, we estimated genetic correlations between SI and 17 metabolic and cardiovascular diseases (Fig. 5A, Table S14 and Table S15). Of the 51 trait–SI pairs examined, 30 (58.8%) showed significant genetic correlations after Bonferroni correction. SI exhibited predominantly negative genetic correlations (rg < 0) with cardiovascular and metabolic diseases, including atrial fibrillation, varicose veins, coronary heart disease, heart failure, hypertension, metabolic syndrome, myocardial infarction, obesity, and type 2 diabetes. The strongest inverse correlations were observed for heart failure (rg = −0.19, *p* = 2.30 × 10^− 9^) and metabolic syndrome (rg = −0.12, *p* = 8.49 × 10^− 8^). These findings suggest that genetic factors associated with a lower risk of sarcopenia, as reflected by higher SI, partially overlap with those conferring reduced susceptibility to cardiometabolic diseases. In contrast, SI showed a significant positive genetic correlation with chronic kidney disease (CKD, rg = 0.38, *p* = 4.67 × 10^− 7^), suggesting a distinct pattern of shared genetic architecture between SI and CKD, potentially reflecting the renal component of this composite biomarker. Notably, sex-stratified analyses revealed that genetic correlations with coronary artery disease were observed only for female SI, whereas associations with hypertension and obesity were specific to male SI. To further investigate shared genetic loci between SI and metabolic traits, as well as sex differences, we extracted independent SI loci identified in males and females and performed cross-phenotype colocalization analyses with 11 metabolic diseases (Figure S7 and Table S16). In total, 276 SI–metabolic trait associations were identified, involving 112 unique SI loci that were associated with at least one metabolic trait. Among these loci, rs10849913 at chr12q24.11 showed the strongest pleiotropic signal, corresponding to 14 SI–metabolic trait associations, with both male and female SI jointly associated with seven metabolic traits. Associations between SI and chronic kidney disease were the most extensive, accounting for 77 associations, followed by type 2 diabetes with 45 associations. Overall, male SI exhibited a greater number of associations with metabolic traits than female SI, with 151 and 125 associations, respectively, indicating stronger shared genetic architecture in males. Notably, two loci, rs1229984 at chr4q23 and rs9817452 at chr3q25.31, were associated exclusively with four metabolic traits exclusively in the male SI analysis (Fig. 5B).

## Discussion

As a major global health concern, sarcopenia substantially impairs quality of life in older adults and increases the risk of adverse health outcomes. In this study, we conducted the first large-scale GWAS of SI to investigate the genetic architecture underlying sarcopenia. Given the pronounced biological differences in muscle mass, strength, and function between males and females, we further performed sex-stratified GWAS analyses, as combined-sex analyses may obscure genetic signals with sex-stratified or sex-dependent patterns.

We observed that the SI exhibited substantial heritability, with an estimated value of 16.2% in the combined-sex analysis. Compared with three commonly used sarcopenia-related traits, ALM, HGS, and WP, which showed heritability estimates of 33.4%, 10.0%, and 7.5%, respectively, the heritability of SI was lower than that of ALM but exceeded that of functional indicators. This intermediate heritability positions SI between structural and functional measures of sarcopenia, suggesting that it captures intrinsic muscle-related biological variation while remaining less influenced by behavioral and environmental factors than functional traits. Consistent with this interpretation, nearly half of the SI-associated loci identified in our analysis were novel, supporting SI as a genetically distinct phenotype capable of revealing genetic mechanisms not detected by conventional sarcopenia indicators.

Given the pronounced differences in muscle biology, hormone regulation, and sarcopenia progression between males and females, combined-sex GWAS that adjust for sex as a covariate may obscure genetic signals with sex-dependent effects. Consistent with this premise, our sex-stratified GWAS identified 135 female-only significant loci and 112 male-only significant loci, including 25 loci that reached genome-wide significance only in sex-stratified analyses. These single-sex significant loci were interpreted as discovery signals rather than definitive evidence of sex-dependent effects unless supported by SDE or gene–sex interaction analyses. Among these loci, rs1145093 at chr15q21.1 within GATM showed particularly strong evidence of sex-differentiated genetic effects, supported by both SDE and genotype–sex interaction analyses, with the latter yielding a highly significant interaction signal (*p* = 1.26 × 10^−2 7^). GATM encodes glycine amidinotransferase, an enzyme involved in creatine biosynthesis together with GAMT. Creatine and phosphocreatine play central roles in cellular energy buffering, particularly in energy-demanding tissues such as skeletal muscle, while creatinine is generated from creatine metabolism [37, 38]. Therefore, genetic variation near GATM provides a biologically plausible link between creatine metabolism and muscle energy homeostasis. Fine-mapping of the GATM region revealed both shared causal variants (rs675876 and rs68087211) and sex-differentiated variants, indicating that sex-dependent effects may arise from a combination of shared genetic architecture and sex-related regulatory mechanisms rather than entirely distinct loci. Consistently, allele frequency differences were excluded as the primary driver of sex heterogeneity. Previous studies have reported sex differences in creatine metabolism and endogenous creatine stores [39], suggesting that creatine/creatinine-related traits may be influenced differently in males and females.

Further genetic analyses identified genes exhibiting sex-biased evidence for association with SI. In males, KLF5 emerged as a prominent male-biased gene. KLF5 is upregulated during early muscle atrophy and cooperates with FOXO1 to induce E3 ubiquitin ligases such as FBXO32, thereby promoting protein degradation through the ubiquitin–proteasome system [40, 41]. This suggests that genetic susceptibility in males may be linked to an accelerated activation of muscle catabolic pathways. In addition to KLF5, male-biased signals included FGF7, which supports muscle satellite cell proliferation and regeneration [42], and MAP1LC3A, a key component of autophagosome formation [43]. Together, these findings indicate that genetic risk in males may arise from enhanced muscle protein breakdown accompanied by dysregulation of regenerative capacity and autophagic processes, ultimately disrupting muscle homeostasis. In females, female-biased associations highlighted distinct biological mechanisms. COQ10A points to mitochondrial dysfunction as a key contributor to genetic susceptibility, potentially exacerbated by postmenopausal declines in estrogen that reduce mitochondrial protection and metabolic resilience [44]. Additionally, NOTUM, a secreted antagonist of Wnt signaling, may impair muscle stem cell activation and tissue repair [45]. These findings suggest that female-biased genetic susceptibility for sarcopenia may be driven primarily by impaired mitochondrial energy metabolism and altered regulation of muscle stem cell function. Enrichment analysis of NR motifs and TFBS in sex-biased gene promoters revealed regulatory mechanisms underlying sex-differentiated regulatory patterns. In males, promoter regions of sex-biased genes showed significant enrichment of AR binding sites, linking genetic susceptibility to male sarcopenia with androgen signaling. Variants affecting AR binding may alter androgen-mediated regulation of muscle growth and protein turnover. PPAR enrichment in male-biased genes further highlighted the importance of lipid oxidation and energy metabolism in male muscle biology [46–48]. Additional TFBS enrichment for GATA4 and IRF3 in male-only significant loci suggested contributions from aging-related transcriptional programs [49] and immune or inflammatory stress responses [50]. In females, SI-associated loci were enriched for ESR1 binding sites, emphasizing estrogen signaling as a key determinant of female genetic risk. Enrichment of PPARα, RXR, and ERRα further implicated lipid metabolism and mitochondrial bioenergetics in female muscle health [48]. Differential enrichment patterns for HIF1A and AR indicated sex-differentiated regulation of hypoxic stress responses and muscle repair. Notably, TFBS enrichment for FOXA2 across sex groups suggested a shared regulatory role in modulating inflammatory responses and maintaining muscle homeostasis.

Pathway enrichment analysis of credible gene sets across sex groups revealed both shared core mechanisms and sex-stratified pathways underlying sarcopenia. In the combined-sex analysis, significant enrichment of the MAPK signaling and cellular senescence pathways confirmed that chronic inflammation, cellular stress, and senescence constitute a common genetic basis of sarcopenia. Sex-stratified gene-set analyses uncovered distinct biological patterns. In females, enriched pathways were primarily related to insulin-like growth factor transport and uptake as well as muscle adaptation, suggesting that genetic susceptibility may be driven by impaired anabolic signaling and reduced responsiveness to growth factors such as IGF-1. Disruption of IGF-mediated signaling may limit muscle protein synthesis and remodeling, contributing to anabolic resistance. In males, enriched pathways were mainly associated with cell migration, regeneration, and development, including Wnt and Sema4D signaling. These pathways are central to muscle stem cell function, tissue repair, and neuromuscular maintenance, indicating that genetic risk in males may be linked to impaired regenerative capacity and compromised neuromuscular integrity, consistent with age-related declines in muscle regeneration [51].

Genetic association analyses revealed significant genetic correlations between the sarcopenia and a broad range of metabolic and cardiovascular diseases. Predominantly negative genetic correlations indicated that variants associated with higher SI values, reflecting better muscle health, were concurrently linked to reduced genetic risk of cardiometabolic disorders. These findings provide genetic support for the role of skeletal muscle as a key metabolic endocrine organ that contributes to systemic metabolic homeostasis. In contrast, SI showed a significant positive genetic correlation with CKD, suggesting shared genetic determinants that influence both traits. Given the biomarker composition of SI, which is derived from serum creatinine and cystatin C, this correlation may partly reflect shared biomarker-related genetic effects rather than a causal effect of higher SI on CKD, while also being consistent with biological crosstalk between kidney function and skeletal muscle [52]. Further colocalization analyses identified a colocalized genetic signal at rs1229984 in the ADH1B locus between male SI and metabolic traits. Although ADH1B is mostly known for its role in alcohol metabolism [53–55], its colocalization with multiple metabolic traits in the male SI analysis, together with reported associations independent of alcohol consumption, suggests alternative mechanisms linking this locus to muscle health. Notably, ADH1B and neighboring genes have been implicated in lipid metabolism and fat distribution, indicating that pleiotropic effects at this locus may influence male SI through dysregulation of lipid and energy balance, potentially shaped by sex-related patterns of adiposity and hormonal regulation.

In summary, this study presents the first large-scale GWAS of SI, confirming previously reported sarcopenia-related loci while identifying novel genetic signals. Our findings highlight sex-stratified genetic patterns, a robust sex-dependent GATM signal, and regulatory differences, and shared genetic architecture between sarcopenia and metabolic diseases. Together, these results provide a genetic framework for improved sarcopenia risk stratification and support future investigation of sex-informed preventive strategies and targeted therapeutic interventions. Several limitations should be acknowledged. First, our analyses were primarily conducted in individuals of white British ancestry, which may limit the generalizability of the findings to other populations. Second, although SI is a practical and accessible biomarker, it may be influenced by diet, medication use, and biomarker-related genetic effects, particularly those related to cystatin C, and therefore should not be interpreted as exclusively reflecting muscle-related biology. In addition, our focus on common genetic variants may have underestimated the contribution of rare variants to sarcopenia risk. Future studies should validate these findings in diverse, multi-ethnic cohorts and integrate rare variant analyses, environmental exposures, and gene–environment interactions to further refine the genetic architecture of sarcopenia and enhance the precision of prevention and treatment strategies.

## Conclusion

Our study provides a large-scale genome-wide investigation of SI and reveals both shared and sex-stratified genetic architectures underlying this accessible biomarker of muscle health. We identified multiple SI-associated loci, with a prominent sex-dependent signal at the rs1145093–chr15q21.1–GATM locus. Further analyses suggested that SI may be influenced by distinct mechanisms in males and females, involving insulin-like growth factor transport and uptake, muscle adaptation, cell migration, regeneration and development. Genetic correlation and cross-phenotype analyses connected SI with cardiometabolic diseases and showed more extensive colocalized associations between male SI and metabolic traits than between female SI and metabolic traits. These findings expand current understanding of SI and underscore the importance of incorporating sex differences into genetic studies of muscle health. Future studies in diverse populations are needed to validate and extend these findings.

## Electronic supplementary material

Below is the link to the electronic supplementary material.

Supplementary Material 1

Supplementary Material 2

## Data Availability

The original data and analytical methods used in this study are publicly available. All analytical methods and detailed procedures used in this study are publicly available through the original source. The GWAS summary statistics generated in this study are publicly available in the GWAS Catalog under accession numbers GCST90832978~GCST90832980.

## Abbreviations

SI: Sarcopenia index
GWAS: Genome-wide association study
ALM: Appendicular lean mass
WP: Walking pace
HGS: Handgrip strength
UKB: UK Biobank
MAF: Minor allele frequency
SNP: Single-nucleotide polymorphism
1kGP3: 1000 Genomes Project Phase 3
LD: Linkage disequilibrium
SDE: Sexually dimorphic effect
PC: Principal component
PIP: Posterior inclusion probability
GTEx: Genotype-Tissue Expression
eQTL: Expression quantitative trait locus
PP.H4: Posterior probability of hypothesis 4
NR: Nuclear receptor
AR: Androgen receptor
TFBS: Transcription factor binding site
LOLA: Locus Overlap Analysis
TF: Transcription factor
KEGG: Kyoto Encyclopedia of Genes and Genomes
GO: Gene Ontology
UTR: Untranslated region
CKD: Chronic kidney disease

## Code availability

The code in this study has been uploaded to https://github.com/binzhi0710/SIGWAS.
