# Supplementary material for: Genome-wide association study of sarcopenia index reveals sex-stratified genetic architecture
Source: Biol Sex Differ. 2026 Aug 25;17:150. doi: 10.1186/s13293-026-00973-y (PMC13548639; doi:10.1186/s13293-026-00973-y)
Supplement: Supplementary file 1 [file 13293_2026_973_MOESM1_ESM.pdf]

# Supplementary Figures

**A**

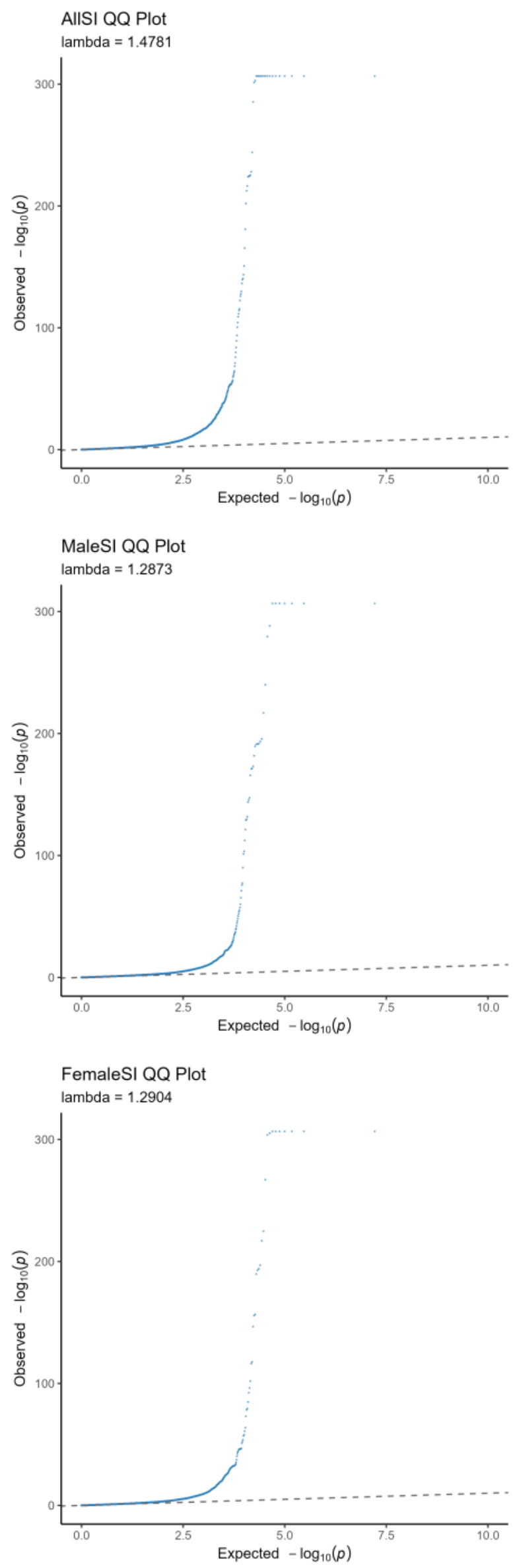


**B**

| Index | Combined-sex | Male | Female |
|---|---|---|---|
| Mean $\chi^2$ | 2.306 | 1.7006 | 1.7273 |
| Intercept | 1.111 | 1.0498 | 1.052 |
| Ratio | 0.085 | 0.0711 | 0.0714 |
| $h^2$ | 0.1617 | 0.1783 | 0.1882 |

**Figure S1 Evidence for the highly polygenic architecture of SI from QQ plots and LD score regression (LDSC) analyses. A** QQ plots for SI in combined-sex, male-specific and female-specific analyses. All plots show marked deviation from the null expectation in the tail, with genomic inflation factors (λ) of 1.4781 (combined), 1.2873 (male), and 1.2904 (female). **B** LDSC results summarizing mean $\chi^2$, intercept, ratio, and SNP-based heritability ($h^2$), indicating an excess of true association signals.

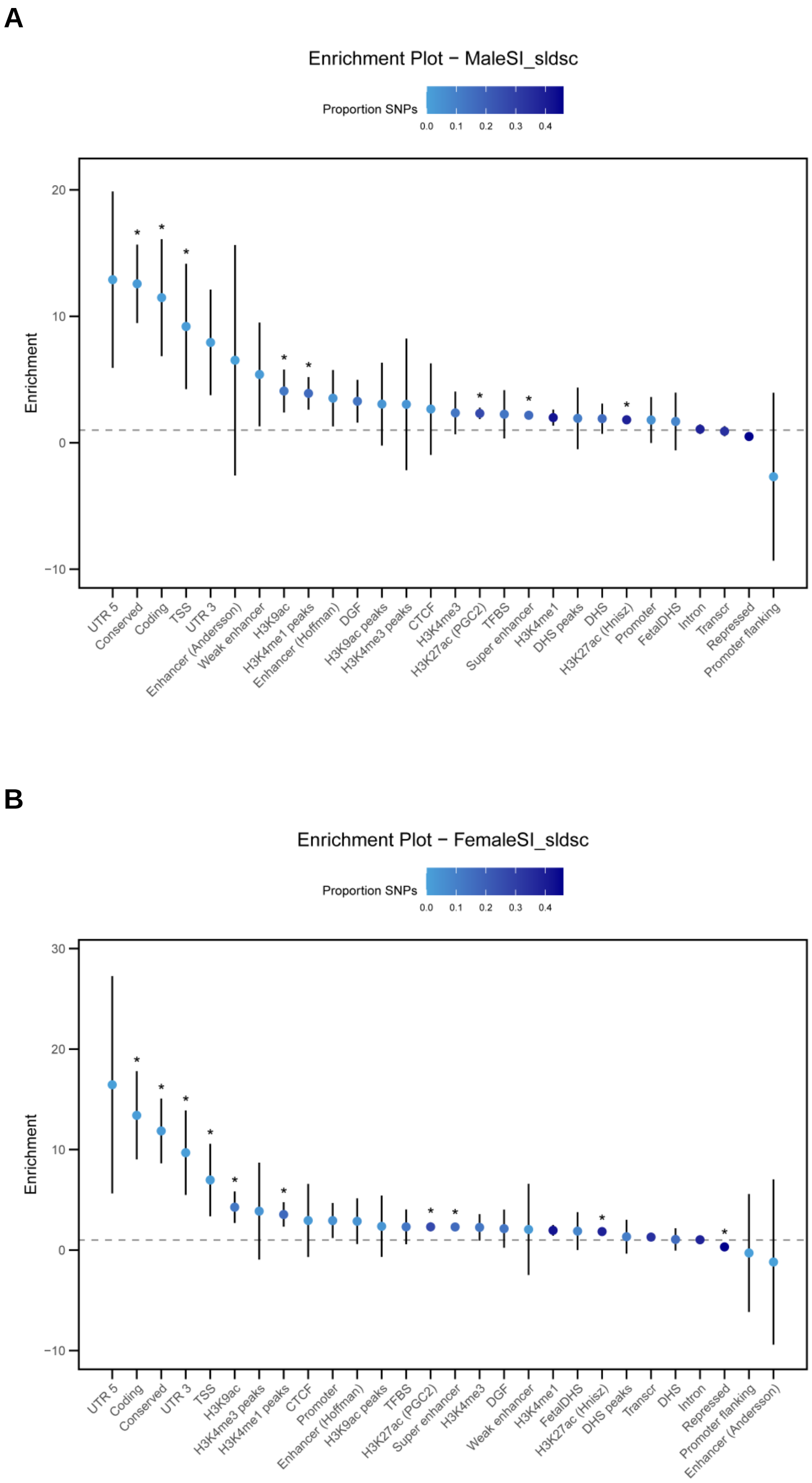


**Figure S2 Partitioned heritability enrichment of SI across functional genomic annotations. A** S-LDSC enrichment analysis for male-specific SI across diverse functional genomic categories. Points represent enrichment estimates with error bars indicating standard errors, and colors denote the proportion of SNPs in each annotation. **B** S-LDSC enrichment analysis for female-specific SI across diverse functional genomic categories.

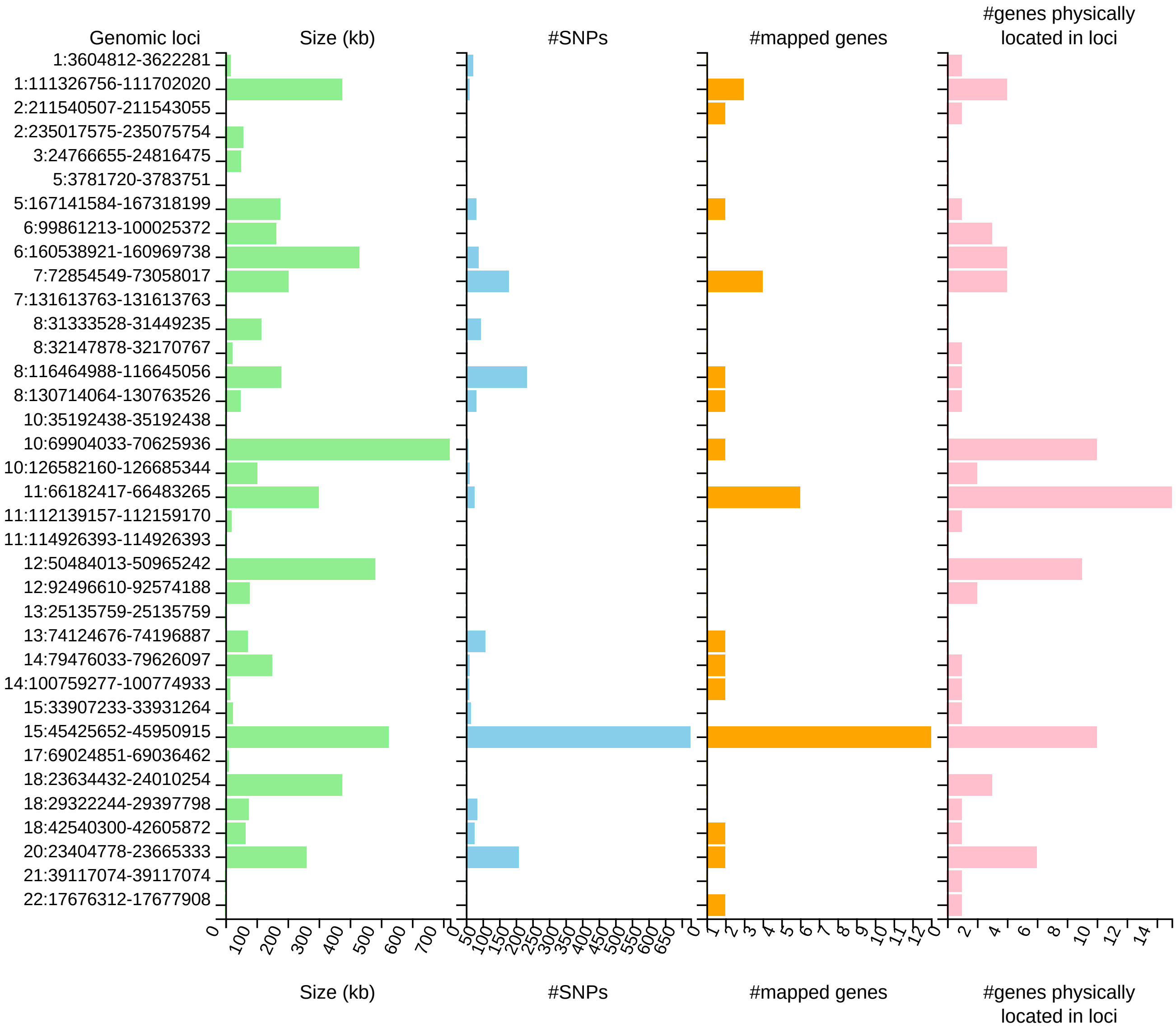


**Figure S3 SI-associated loci identified by SDE analysis.** Genomic loci with pval<$1\times10^{-5}$ were displayed. For each locus, four features are displayed: locus size (kb), number of associated SNPs, number of mapped genes, and number of genes physically located within the locus.

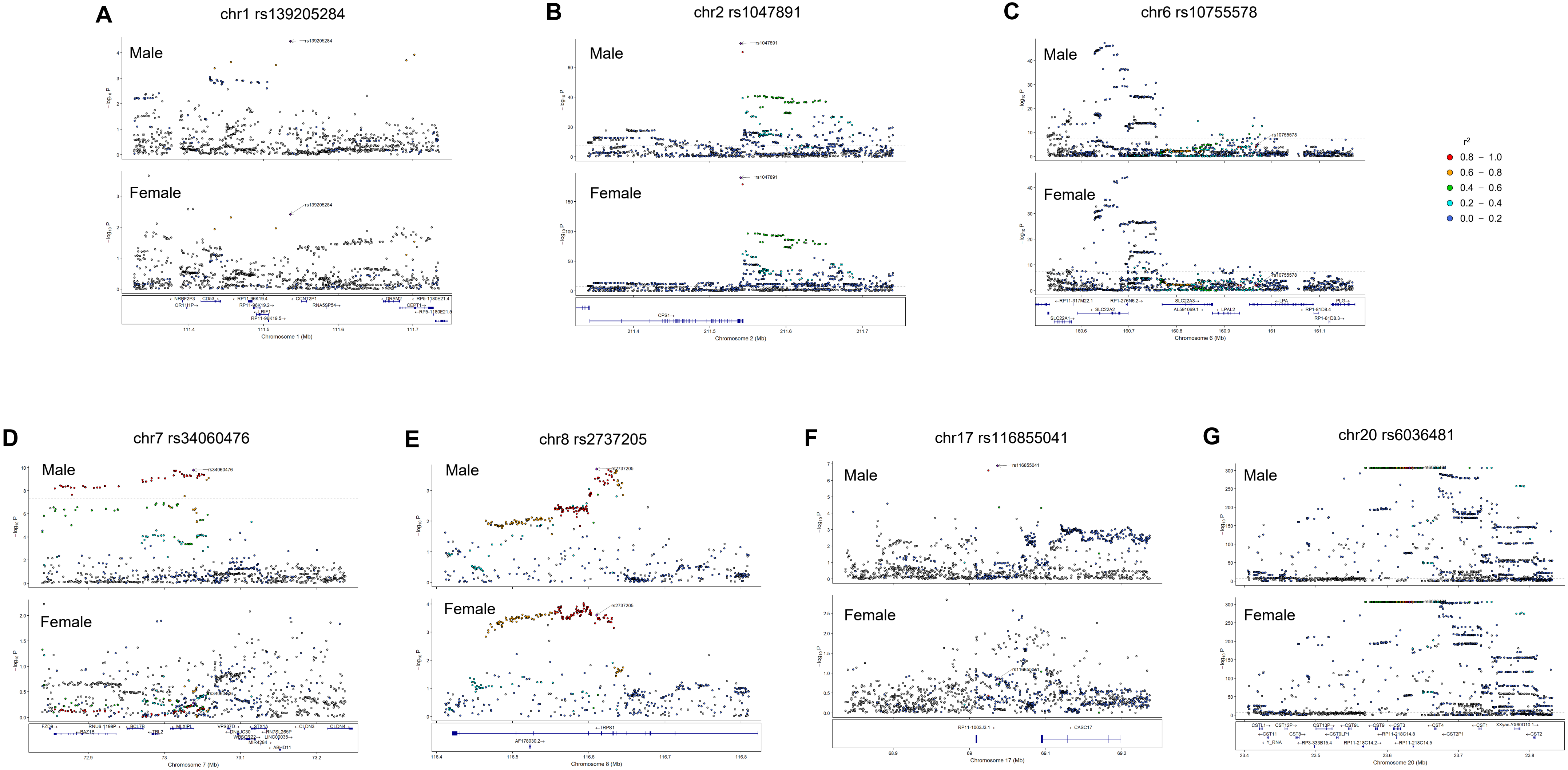


**Figure S4 Regional association plots for sex-differential effect loci with P < 1 × 10$^{-6}$, excluding the GATM locus.** For each locus, the upper panel shows the regional association pattern in males and the lower panel shows the corresponding pattern in females. The gene track below each locus indicates the genomic positions and orientations of nearby genes.

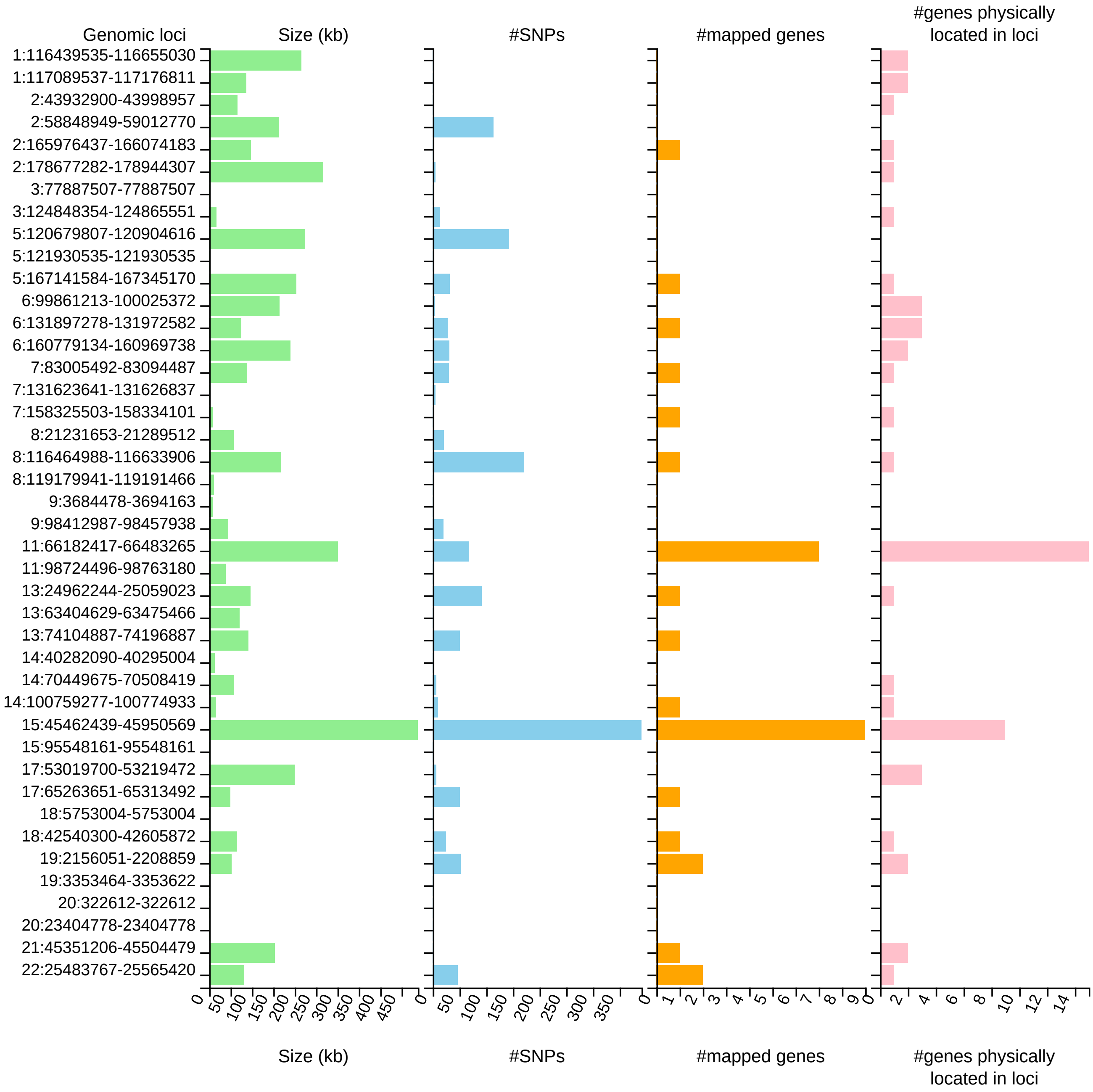


**Figure S5 SI-associated loci identified by gene-sex interaction analysis.** Genomic loci with pval<$1\times10^{-5}$ were displayed. For each locus, four features are displayed: locus size (kb), number of associated SNPs, number of mapped genes, and number of genes physically located within the locus.

**A**

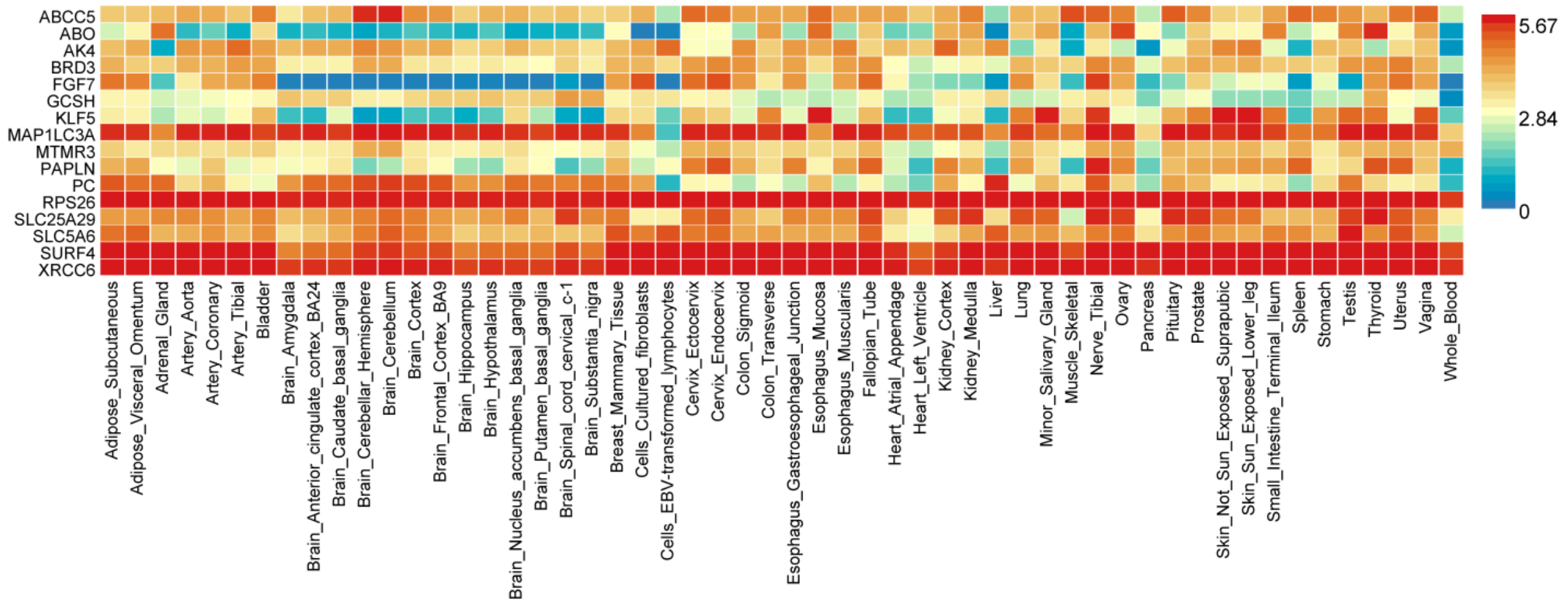


**B**

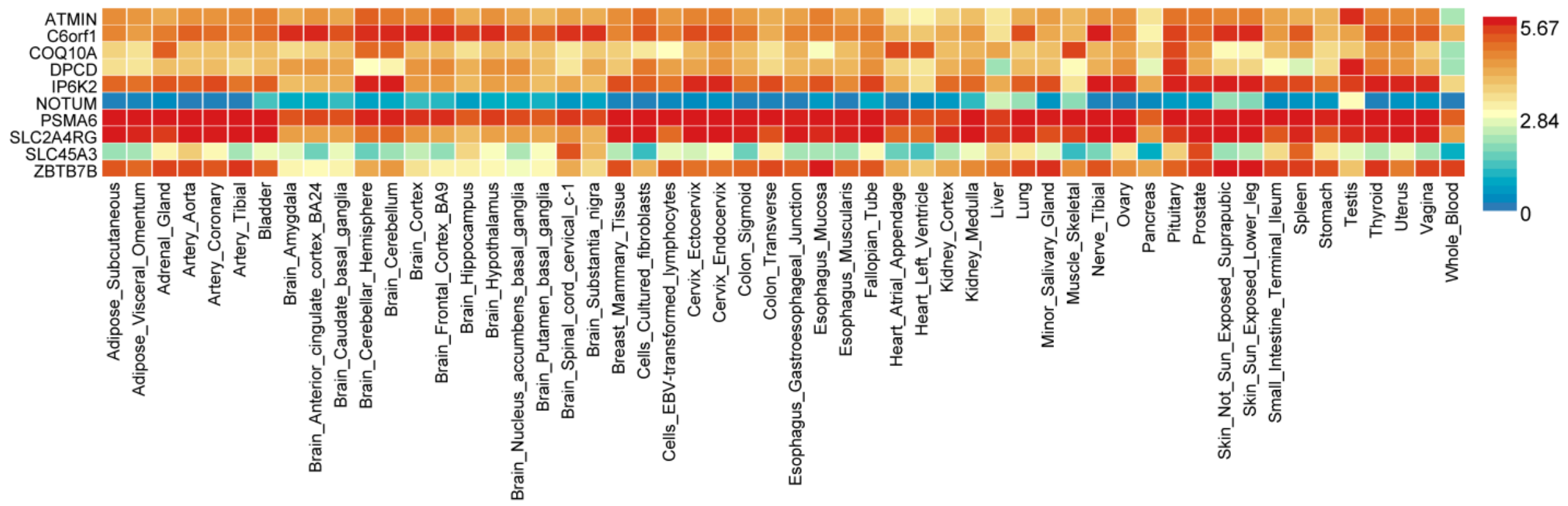


**Figure S6 Tissue-specific expression profiles of sex-specific genes across GTEx v8 tissues. A** Heatmap showing the expression levels of male-specific genes across all GTEx v8 tissues. **B** Heatmap showing the expression levels of female-specific genes across all GTEx v8 tissues.

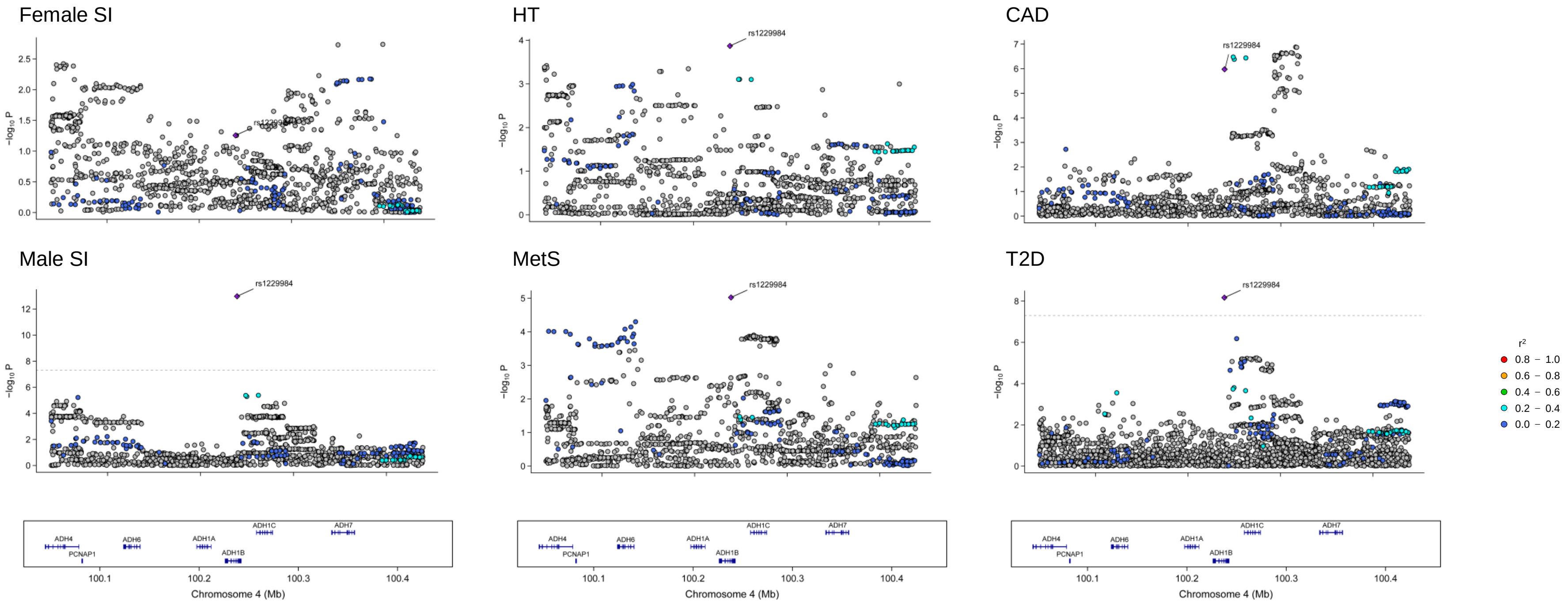


**Figure S7 Colocalization analysis of SI-associated loci.** Regional association plots for female SI, male SI, and cardiometabolic traits (HT, CAD, MetS, and T2D) on chromosome 4. SNPs are plotted by genomic position and $-\log_{10}(p)$, with colors indicating linkage disequilibrium ($r^2$) with the lead variant rs1229984. SI, sarcopenia index; HT, hypertension; CAD, coronary artery disease; MetS, metabolic syndrome; T2D, type 2 diabetes.
